# Modest Effects of Osteoclast‐Specific ERα Deletion after Skeletal Maturity

**DOI:** 10.1002/jbm4.10797

**Published:** 2023-07-13

**Authors:** Madison L. Doolittle, Brittany A. Eckhardt, Stephanie J. Vos, Sarah Grain, Jennifer L. Rowsey, Ming Ruan, Dominik Saul, Joshua N. Farr, Megan M. Weivoda, Sundeep Khosla, David G. Monroe

**Affiliations:** ^1^ Robert and Arlene Kogod Center on Aging and Division of Endocrinology Mayo Clinic College of Medicine Rochester Minnesota USA; ^2^ Department of Trauma and Reconstructive Surgery Eberhard Karls University Tübingen, BG Trauma Center Tübingen Tübingen Germany; ^3^ Robert and Arlene Kogod Center on Aging and Division of Hematology Mayo Clinic College of Medicine Rochester Minnesota USA

**Keywords:** ESTROGENS AND SELECTIVE ESTROGEN RECEPTOR MODULATOR, GENETIC ANIMAL MODELS, OSTEOCLASTS, OSTEOPOROSIS, SEX STEROIDS

## Abstract

Estrogen regulates bone mass in women and men, but the underlying cellular mechanisms of estrogen action on bone remain unclear. Although both estrogen receptor (ER)α and ERβ are expressed in bone cells, ERα is the dominant receptor for skeletal estrogen action. Previous studies using either global or cell‐specific ERα deletion provided important insights, but each of these approaches had limitations. Specifically, either high circulating sex steroid levels in global ERα knockout mice or the effects of deletion of ERα during growth and development in constitutive cell‐specific knockout mice have made it difficult to clearly define the role of ERα in specific cell types in the adult skeleton. We recently generated and characterized mice with tamoxifen‐inducible ERα deletion in osteocytes driven by the 8‐kb Dmp1 promoter (ERαΔOcy mice), revealing detrimental effects of osteocyte‐specific ERα deletion on trabecular bone volume (−20.1%) and bone formation rate (−18.9%) in female, but not male, mice. Here, we developed and characterized analogous mice with inducible ERα deletion in osteoclasts using the *Cathepsin K* promoter (ERαΔOcl mice). In a study design identical to that with the previously described ERαΔOcy mice, adult female, but not male, ERαΔOcl mice showed a borderline (−10.2%, *p* = 0.084) reduction in trabecular bone volume, no change in osteoclast numbers, but a significant increase in serum CTx levels, consistent with increased osteoclast activity. These findings in ERαΔOcl mice differ from previous studies of constitutive osteoclast‐specific ERα deletion, which led to clear deficits in trabecular bone and increased osteoclast numbers. Collectively, these data indicate that in adult mice, estrogen action in the osteocyte is likely more important than via the osteoclast and that ERα deletion in osteoclasts from conception onward has more dramatic skeletal effects than inducible osteoclastic ERα deletion in adult mice. © 2023 The Authors. *JBMR Plus* published by Wiley Periodicals LLC on behalf of American Society for Bone and Mineral Research.

## Graphical abstract

Application of the newly developed *Ctsk‐CreERT2* genetic mouse model permits inducible deletion of estrogen receptor alpha (ERα) in osteoclasts through administration of tamoxifen (Tam). While constitutive deletion of ERα from conception onward results in decreased BV/TV and increased osteoclast numbers, inducible deletion at skeletal maturity results in no significant change in these bone parameters, however results in increased CTx (a marker for osteoclast activity).

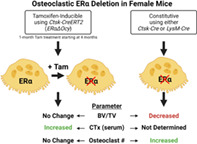

## Introduction

Although estrogen has been shown to be the major sex steroid regulating bone mass in women and men,^(^ ^1^ ^)^ the cellular targets and underlying mechanisms of estrogen action on bone remain to be fully defined. As in other tissues, bone cells express both estrogen receptor (ER)α and ERβ, but ERα appears to be the dominant receptor regulating bone metabolism.^(^ ^2^, ^3^ ^)^ Different groups have assessed the skeletal consequences of deleting ERα both globally and specifically in osteoprogenitors,^(^ ^4^ ^)^ osteoblasts/osteocytes,^(^ ^5^, ^6^, ^7^ ^)^ osteoclasts,^(^ ^8^, ^9^ ^)^ and immune cells.^(^ ^10^ ^)^ Although these models are informative, a potential problem with them is that the deletion of ERα occurs from conception onward. Because postmenopausal osteoporosis is caused by a loss of estrogen signaling in adulthood, after the skeleton has fully matured, the relevance of these previous studies is difficult to translate to human physiology.

To address this problem, we previously developed and characterized mice with inducible ERα deletion in osteocytes using the 8‐kb Dmp1 promoter.^(^ ^11^ ^)^ This model demonstrated important similarities and differences in skeletal phenotype compared to mice with constitutive osteocytic ERα deletion.^(^ ^5^, ^6^, ^7^ ^)^ Here, we develop and phenotype mice with inducible ERα deletion specifically in osteoclasts using the Cathepsin K (*Ctsk*) promoter. We then contrast the skeletal phenotypes of these mice to our previous osteocyte‐specific inducible model,^(^ ^5^, ^6^, ^7^ ^)^ as well as to the previously described models of osteoclast‐specific constitutive ERα deletion.^(^ ^8^, ^9^ ^)^

## Materials and Methods

### ERα^fl^ ^/fl^ and TdTomato mice

ERα^fl/fl^ mice were previously described and characterized.^(^ ^11^, ^12^ ^)^ In these mice, exon 3 of the mouse ERα is flanked by lox P recombination sites. All mice used in this study, including these mice, were in the C57BL/6 background. The TdTomato mouse strain (B6;129S6‐Gt(ROSA)26Sor^tm9(CAG‐tdTomato)Hze^/*JAi9*)^(^ ^13^ ^)^ was obtained from Jackson Laboratory under stock no. 007905.

### Ctsk‐CreERT2 construct design and transgenic mouse production

We developed an inducible osteoclast‐specific Cre model using a validated *Ctsk* promoter described previously by the Davey group, which has been shown to have high specificity for osteoclasts.^(^ ^14^ ^)^ This promoter consists of nucleotides −3359 to +1660 of the *Ctsk* gene.^(^ ^14^ ^)^ The *Ctsk‐CreERT2* construct was made by polymerase chain reaction (PCR) amplifying 5.1 kb of the *Ctsk* promoter in mouse genomic DNA using LongAmp *Taq* DNA Polymerase (New England Biolabs, Ipswich, MA, USA). This product was blunt‐end cloned into the Pme1 and HpaI sites of the *attB*‐containing pBT378 plasmid,^(^ ^15^ ^)^ with a 3′ MluI site incorporated to facilitate the next cloning step. The CreERT2 gene was PCR amplified from *pCAG‐CreERT2* (Addgene, Watertown, MA, USA) and cloned into this 3′ MluI site to produce the final *Ctsk‐CreERT2* construct. Transgenic mice were produced through the Stanford Transgenic, Knockout and Tumor Model Center by selectively inserting the *Ctsk‐CreERT2* construct into the ROSA locus in C57BL/6 mice using integrase‐mediated transgenesis.^(^ ^15^ ^)^ This technique assures a high efficiency of a single‐copy transgene insertion into a predetermined and transcriptionally active chromosome locus.

### Mouse husbandry and genetic crosses

Animal studies were conducted in accordance with National Institutes of Health (NIH) guidelines and with approval from the Institutional Animal Care and Use Committee (IACUC) at the Mayo Clinic, with all assessments performed in a blinded fashion. We used both male and female adult mice (aged 4 months, collected at 5 months) for our experimental procedures. To generate experimental mice, heterozygous *CtskCreERT2* males were bred with homozygous *ERαfl/fl* females to generate *CtskCreERT2+/−/ERαfl/+* heterozygotes. Sires from this cross were then bred to *ERαfl/+* mice to generate *CtskCreERT2*+/−*/ERαfl/fl* and *CtskCreERT2*+/− groups. All mice phenotyped were littermates.

### Tamoxifen treatments

Tamoxifen (Sigma‐Aldrich, St. Louis, MO, USA) was dissolved in 98% corn oil, 2% ethanol to 10 mg/mL and delivered subcutaneously at a dose of 50 mg/kg. In *TdTomato* experiments, 4‐month‐old *CtskCreERT2* × *TdTomato* mice were injected with either corn oil or tamoxifen for five consecutive days (D1‐5), then intermittently (D15 and D22) before sacrifice (D31). This dosing scheme was similarly used for the experimental phenotyping studies. Due to tamoxifen effects on bone metabolism,^(^ ^16^, ^17^ ^)^ all mice were treated with tamoxifen.

### Tissue collection

Mice were anesthetized (ketamine/xylazine) and blood was collected by cardiac puncture and stored at −80°C. The L4‐L6 lumbar vertebrae were dissected, cleaned, and stored in ethanol for micro–computed tomography (μCT). Right femurs were embedded in methyl methacrylate (MMA) for bone histomorphometry. Both tibiae and the thoracic vertebrae were isolated and cleaned, and marrow‐free bone samples were obtained using centrifugation, as described previously.^(^ ^18^ ^)^ Samples were homogenized in QIAzol Lysis reagent (QIAGEN, Valencia, CA, USA) and stored at −80°C.

### TdTomato histology and staining

Femur, lumbar spine, and soft tissues were fixed in 4% paraformaldehyde (PFA) at 4°C for 72 h under gentle agitation. Bones were decalcified in 10% EDTA for 2 weeks at 4°C under gentle shaking agitation, followed by incubation in 30% sucrose for 3 days at 4°C. Samples were embedded in Cryomatrix (Thermo Fisher Scientific, Wilmington, DE, USA) and flash frozen in liquid nitrogen and stored at −80°C. Cryosections 7 μm thick were prepared for fluorescent imaging. Bone sections were stained for tartrate‐resistant acid phosphatase (TRACP) activity to detect osteoclasts using a fluorescent phosphatase substrate (ELF97).^(^ ^19^ ^)^ ELF97 was dissolved 1:25 in TRACP staining solution (1 mM sodium nitrite, 100 mM acetate, and 7.4 mM tartrate), and sections were stained for 15 min at 37°C. Sections were mounted with ProLong Antifade (Thermo Fisher Scientific, Waltham, MA, USA) and all slides were imaged using the Zeiss Axio Observer Z1 microscope (Carl Zeiss Microscopy, LLC) and ZenPro software (Carl Zeiss Microscopy, LLC).

### RNAscope analyses

In situ hybridization of *ERα* mRNA in osteoclasts was performed on FFPE bone sections (*n* = 4 per group) from the lumbar spine using the RNAScope 2.5 HD Reagent kit (Advanced Cell Diagnostics [ACD], Newark, CA, USA). Then 5‐μm‐thick paraffin sections were deparaffinized, followed by Pepsin Reagent (Sigma) antigen retrieval for 30 min at 37°C. Target probes for *ERα* (*Esr1*) (Catalog No. 478201, ACD) and *Oscar* (Catalog No. 1179641‐C1, ACD) were used with the RNAScope procedure followed according to the manufacturer's instructions. Sections were mounted (VectaMount, Vector Laboratories, Burlingame, CA, USA) and visualized using a ×40 objective of the Nikon Eclipse TI microscope. Approximately 200 osteoclasts were counted per section (in 30 separate fields of view) and scored for *ERα* positivity.

### Quantitative PCR (qPCR) analysis

Total RNA was extracted using RNeasy Mini Columns with DNase solution (QIAGEN). Reverse transcriptase was performed with the High‐Capacity cDNA Reverse Transcription Kit (Applied Biosystems by Life Technologies, Foster City, CA, USA). qRT‐PCR was performed on the ABI Prism 7900HT Real Time System (Applied Biosystems, Carlsbad, CA, USA) using SYBR green (QIAGEN). The mouse primer sequences are provided in Table S1. Input RNA was normalized using housekeeping genes (*Actb*, *Gapdh*, *Hprt*, *Tuba1a*, *Tbp*) from which the most stable reference gene was determined by the geNorm algorithm.^(^ ^20^ ^)^ The delta Ct for each gene was used to calculate the relative mRNA expression changes for each sample. Genes with Ct values above 35 were considered not expressed, as was done previously.^(^ ^21^ ^)^

### Skeletal phenotyping

μCT imaging was performed on a Viva Scan 40 μCT scanner (Scanco Medical AG, Basserdorf, Switzerland) with the following parameters: 55 kVp, 145 mA, high resolution, 21.5 diameter, 10.5‐μm voxel size, 300‐ms integration time. Longitudinal analysis of bone microarchitecture was performed on the tibial diaphysis at baseline (4 months) and endpoint (5 months) before sacrifice. Animals were anesthetized using 2% to 4% isoflurane inhalation for induction and 1% isoflurane for maintenance and remained immobilized for the entirety of the scan. In each mouse, the distal epiphysis of the tibia was identified (specifically the tibia/fibula junction), and diaphysis scans were initiated 1 mm proximal to this anatomical landmark. Cortical parameters were assessed at the tibial midshaft diaphysis (50 slices). Ex vivo quantitative analysis of the lumbar spine (L5) was performed on dissected tissue after sacrifice. Three‐dimensional analysis was used to calculate morphometric parameters at the lumbar vertebral body (200 slices) defining trabecular bone mass and microarchitecture.

### Bone histomorphometry

All histological analyses were done in a blinded fashion. For dynamic histomorphometry, mice were injected subcutaneously with Alizarin‐3‐methyliminodiacetic acid (0.1 mL/animal, 7.5 mg/mL) and calcein (0.1 mL/animal, 2.5 mg/mL) on days 9 and 2, respectively, before euthanasia. Bone sectioning and histomorphometry were performed as previously described.^(^ ^3^ ^)^

### Serum protein measurements

Blood was drawn from cardiac bleeds from overnight fasted mice. Cardiac blood was allowed to clot, and serum was collected by centrifugation at 8500 rpm for 5 min at room temperature. Bone marker assays were conducted for PINP (amino‐terminal propeptide of type I collagen) using the Rat/Mouse PINP enzyme immunoassay (EIA) kit (Immuno Diagnostic Systems [IDS], Scottsdale, AZ, USA) and CTx (cross‐linked C‐telopeptide of type I collagen) using the RatLaps Rat/Mouse CTx EIA kit (IDS). Serum E_2_ was measured by liquid chromatography–mass spectrometry (LC–MS)/MS (API 5000, Applied Biosystems‐MDS Sciex; interassay CV 8%).

### Statistical analyses

Statistically significant differences were determined in Graphpad Prism version 9.3.1 (GraphPad Software, Inc., La Jolla, CA, USA) and R (version 4.0.3). All data were tested for normality using the Shapiro–Wilk test, and for parametric data we performed an unpaired *t*‐test. Experimental group numbers are indicated in each figure. Mouse group sizes were based on previously conducted and published experiments.^(^ ^3^, ^11^ ^)^

## Results

### Construction of osteoclast‐specific Ctsk‐CreERT2 model

**Fig. 1:**
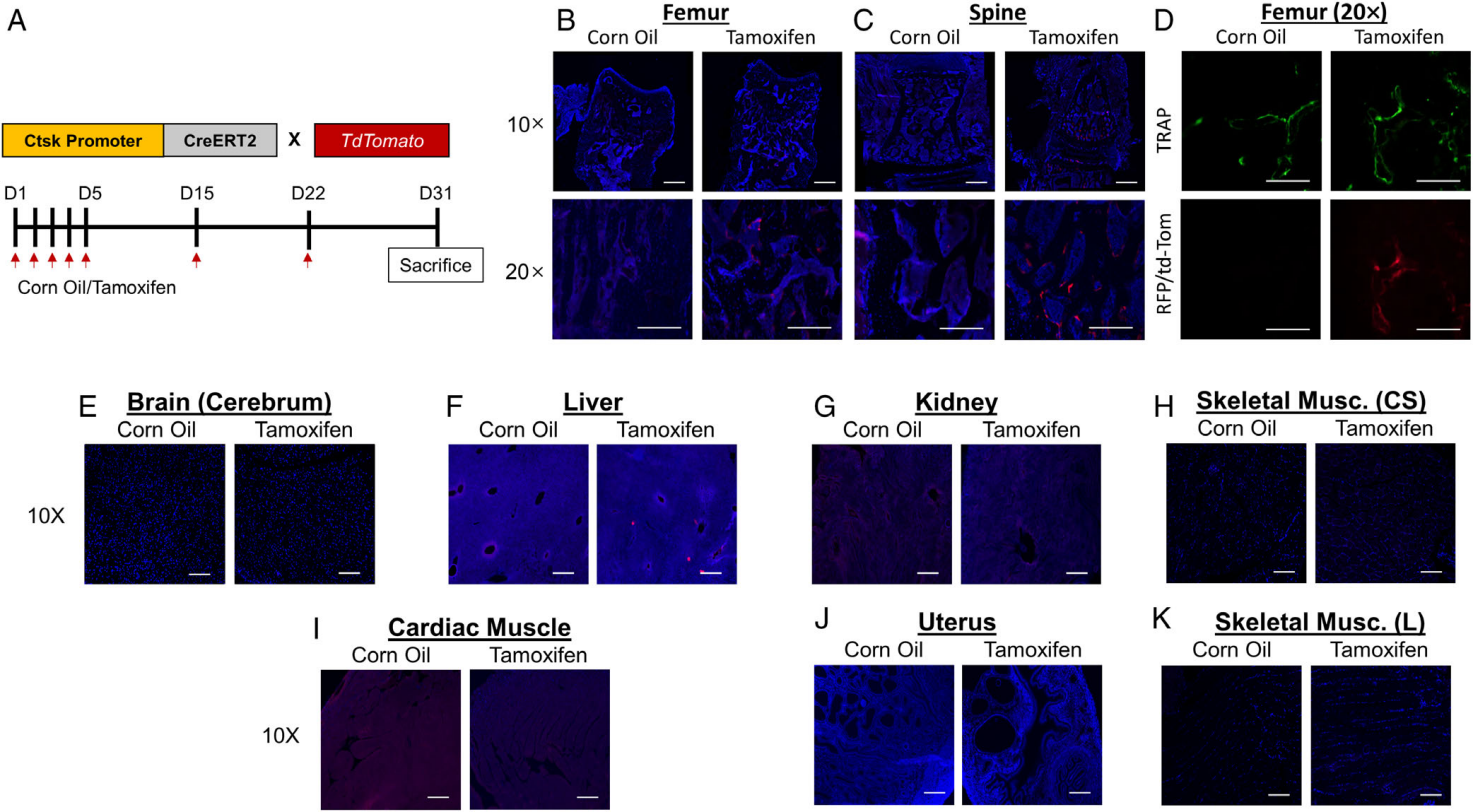
The *Ctsk‐CreERT2* mouse model targets osteoclasts. (*A*) Experimental outline for model validation in *Ctsk‐CreERT* × *TdTomato* mice. (*B*) Representative images of induced TdTomato expression in osteoclasts in femur and (*C*) spine of corn oil‐ versus tamoxifen‐treated *Ctsk‐CreERT2 × TdTomato* mice at low (×10) and high (×20) magnification. (*D*) Fluorescent TRACP staining of femur sections indicating overlap with TdTomato+ cells. (*E*–*L*) Representative images of induced TdTomato expression in various soft tissues of corn oil‐ versus tamoxifen‐treated *Ctsk‐CreERT2* × *TdTomato* mice. CS = cross section; L = longitudinal. Scale bars = 100 μm.

Generation of the *Ctsk‐CreERT2* mice is described in detail in the Methods section. The *Ctsk‐CreERT2* mice were crossed with Ai9 *TdTomato* mice and treated with tamoxifen (Fig. 1*A*) to evaluate specificity for osteoclasts. In the tamoxifen‐treated mice, but not the corn oil‐treated control mice, we observed selective recombination on the surfaces of trabecular bone in the femur and spine (Fig. 1*B*,*C*). For specificity for osteoclasts, we next performed fluorescent TRACP staining, demonstrating colocalization of TRACP and *TdTomato* in the tamoxifen‐ but not in the corn oil‐treated mice (Fig. 1*D*). To evaluate the possible expression of Cre in the *Ctsk‐CreERT2* mice in cells other than osteoclasts, we evaluated a number of other tissues, finding no evidence of Cre expression in brain, kidney, uterus, and skeletal as well as cardiac muscle, with minimal recombination in the liver (Fig. 1*E*–*K*). We also examined femoral bone sections for periosteal Ctsk expression, since these cells arise from the Groove of Ranvier and are Ctsk positive. As seen in Fig. S1, TdTomato signal is only observed on endosteal bone surfaces.

### Validation of Ctsk‐CreERT2‐mediated ERα deletion in adult mice

**Fig. 2:**
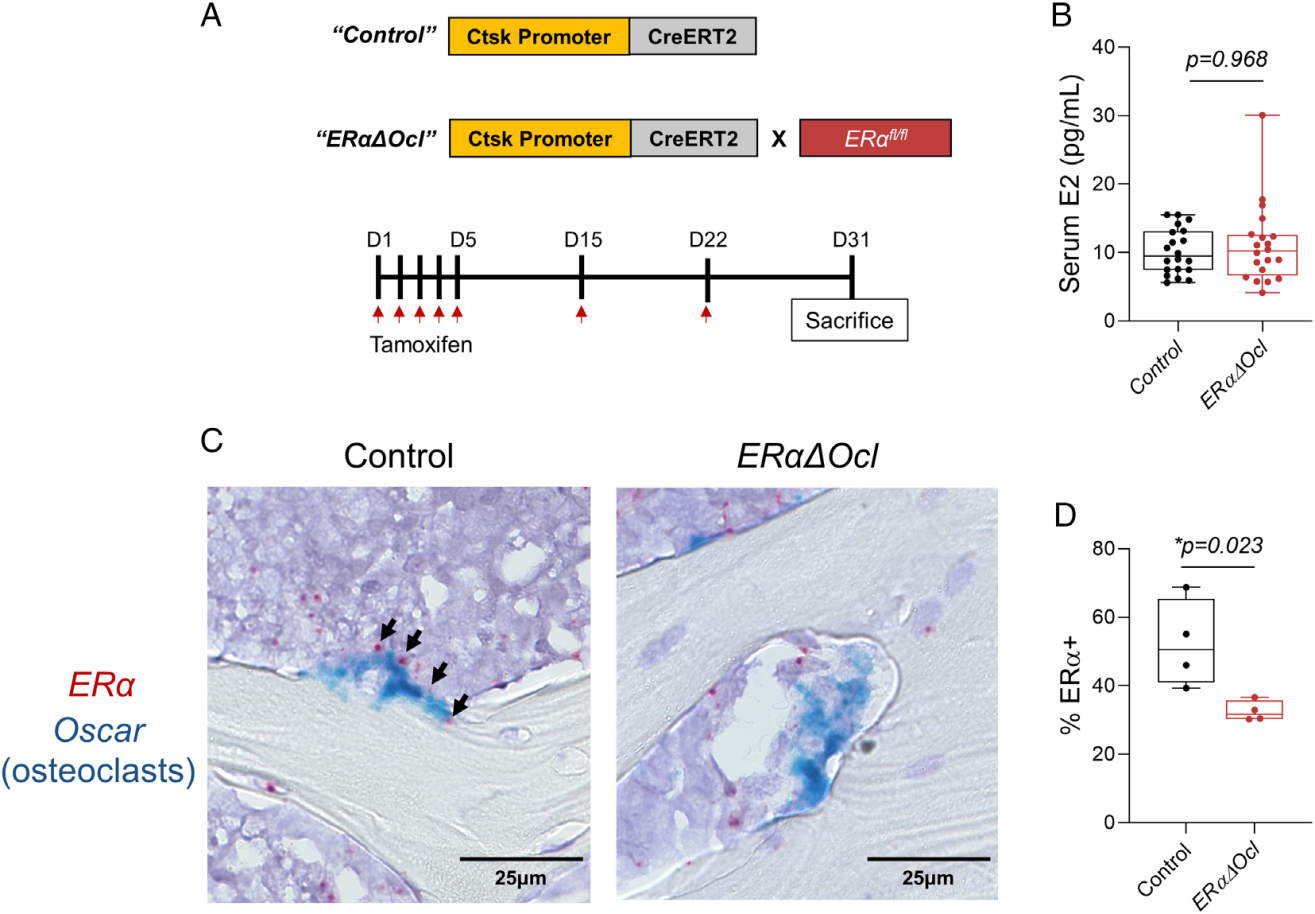
Validation of osteoclast‐specific ERα deletion in adult *ERαΔOcl* mice. (*A*) Experimental outline for inducible deletion of ERα in *Ctsk* + osteoclasts. (*B*) Measurement of serum estradiol (E2) from cardiac blood (*n* = 15–16 per group). (*C*) RNAscope images of *Esr1* (red: encoding ERα) and osteoclast‐specific *Oscar* (blue) mRNA probes performed on spine bone sections from tamoxifen‐treated *ERαΔOcl* mice. Magnification ×40, scale bars = 25 μm. Black arrows point to ERα positivity on osteoclasts. (*D*) Quantification of percentage of *Oscar* + osteoclasts positive for ERα observed from RNAscope (*n* = 4 per group; two male, two female). Statistical significance was determined by unpaired *t*‐test.

We next crossed the *Ctsk‐CreERT2* mice with mice homozygous for a floxed allele of the gene encoding estrogen receptor α (*ERα* ^*fl/fl*^)^(^ ^12^ ^)^ to create mice with inducible deletion of ERα in osteoclasts following tamoxifen treatment (“*ERαΔOcl*” mice; see Methods for breeding strategy) (Fig. 2*A*). Given the known effects of tamoxifen on bone,^(^ ^16^, ^17^ ^)^ we used control mice that were *CreERT2* only, which were treated with identical doses of tamoxifen. Because global deletion of ERα in mice leads to elevated circulating estrogen levels secondary to hypothalamic–pituitary feedback,^(^ ^22^ ^)^ we also measured serum estradiol levels, which were no different in the control versus the *ERαΔOcl* mice (Fig. 2*B*).

Given that osteoclasts constitute only a minority of cells in the bone microenvironment and are relatively difficult to isolate using flow sorting, we used in situ hybridization (RNAScope) to measure *ERα* mRNA along with the osteoclast‐specific marker *Oscar* ^(^ ^23^ ^)^ to demonstrate the efficacy of osteoclast‐specific *ERα* deletion in the *ERαΔOcl* mice. Using this approach, we were able to clearly demonstrate a significant reduction in the *ERα* mRNA in *Oscar*‐expressing osteoclasts (Fig. 2*C*,*D*). Specifically, we found that the percentage of *ERα* + osteoclasts decreased from 52.3 ± 12.7% in the control mice to 32.5 ± 3.0% in the *ERαΔOcl* mice (*p* = 0.023). Although the reduction was modest, we note that its magnitude in *ERα* + osteoclasts in the inducible *ERαΔOcl* mice was almost identical to the reduction of *ERα* + osteocytes that we recently observed in a parallel model for osteocyte‐specific *ERα* deletion using the 8‐kb *Dmp1* promoter (*ERαΔOcy*),^(^ ^11^ ^)^ where we nonetheless observed substantial deficits in trabecular bone in female mice following inducible osteocytic *ERα* deletion.

### Skeletal phenotyping of ERαΔOcl mice

**Fig. 3:**
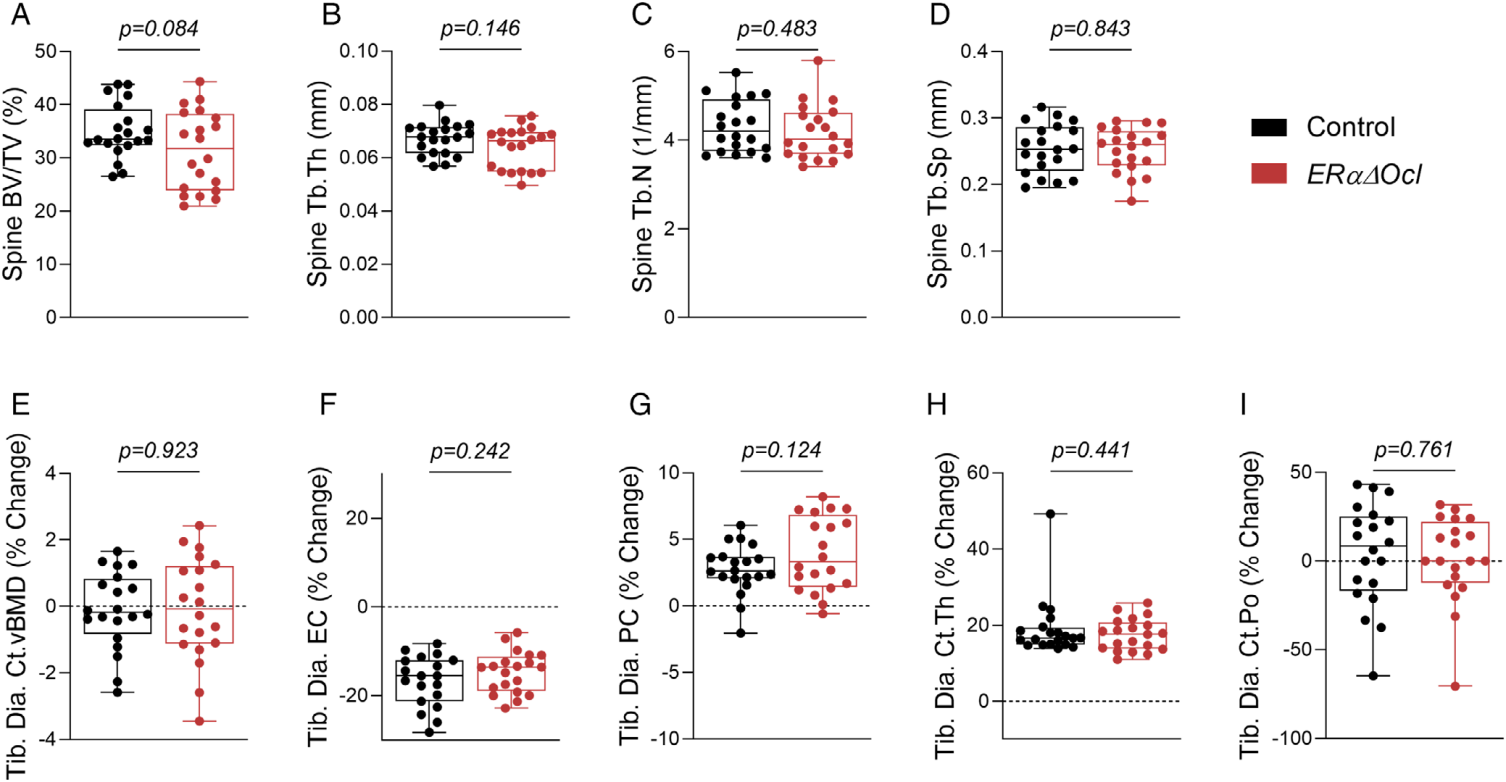
Osteoclast‐specific deletion of ERα in adult female mice does not alter bone mass. μCT analyses of lumbar spine demonstrating (*A*) bone volume fraction (BV/TV), (*B*) trabecular thickness (Tb.Th), (*C*) trabecular number (Tb.N), and (*D*) trabecular spacing (Tb.Sp). (*E*–*I*) Longitudinal μCT analyses of cortical bone at tibial diaphysis (Tib. Dia.) showing percentage (%) change between baseline and endpoint. (*E*) Cortical volumetric BMD (vBMD), (*F*) endocortical circumference (EC), (*G*) periosteal circumference (PC), (*H*) cortical thickness (Ct.Th), and (*I*) cortical porosity (Ct.Po). *n* = 19–20 mice per group. Statistical significance was determined by unpaired *t*‐test.

As noted in the preceding discussion, due to the known effects of tamoxifen on bone,^(^ ^16^, ^17^ ^)^ we compared the skeletal phenotypes of the *ERαΔOcl* mice to control *Ctsk‐CreERT2* mice treated identically with tamoxifen. Four‐month‐old *ERαΔOcl* and control mice were treated with the tamoxifen regimen and assessed for skeletal effects at 5 months of age (Fig. 1*A*). In female *ERαΔOcl* mice, we observed a nonsignificant trend (−10.2%, *p* = 0.084) for a reduction in spine bone volume fraction (BV/TV, Fig. 3*A*), but no other changes in spine trabecular bone (Fig. 3*B*–*D*). Next, we assessed changes in cortical bone parameters through longitudinal μCT of the tibial diaphysis. We found no changes in cortical volumetric BMD (vBMD) or alterations in rates of endocortical or periosteal apposition in the *ERαΔOcl* mice (Fig. 3*E*–*G*), while also observing no change in cortical thickness or porosity (Fig. 3*H*,*I*). Note that tibial endocortical diameter was decreased and periosteal diameter was increased in both the control and *ERαΔOcl* mice, likely reflecting an effect of tamoxifen through ERα in cells other than osteoclasts.

In male mice, we also observed no change in spine trabecular BV/TV or other parameters in *ERαΔOcl* mice (Fig. S2 *A*,*D*), other than a minor increase in spine trabecular thickness (Tb.Th) and a concordant reduction in trabecular spacing (Tb.Sp) (Fig. S2 *B*,*D*). Interestingly, in male *ERαΔOcl* mice relative to control mice, we observed endocortical surface expansion in the tibia, in addition to reduced overall cortical thickness as measured by longitudinal μCT (Fig. S1 *E*,*F*). This was accompanied by no change in periosteal surface diameter, cortical vBMD, or cortical porosity (Fig. S1 *G*–*I*).

### Alterations in cellular function in ERαΔOcl mice

**Fig. 4:**
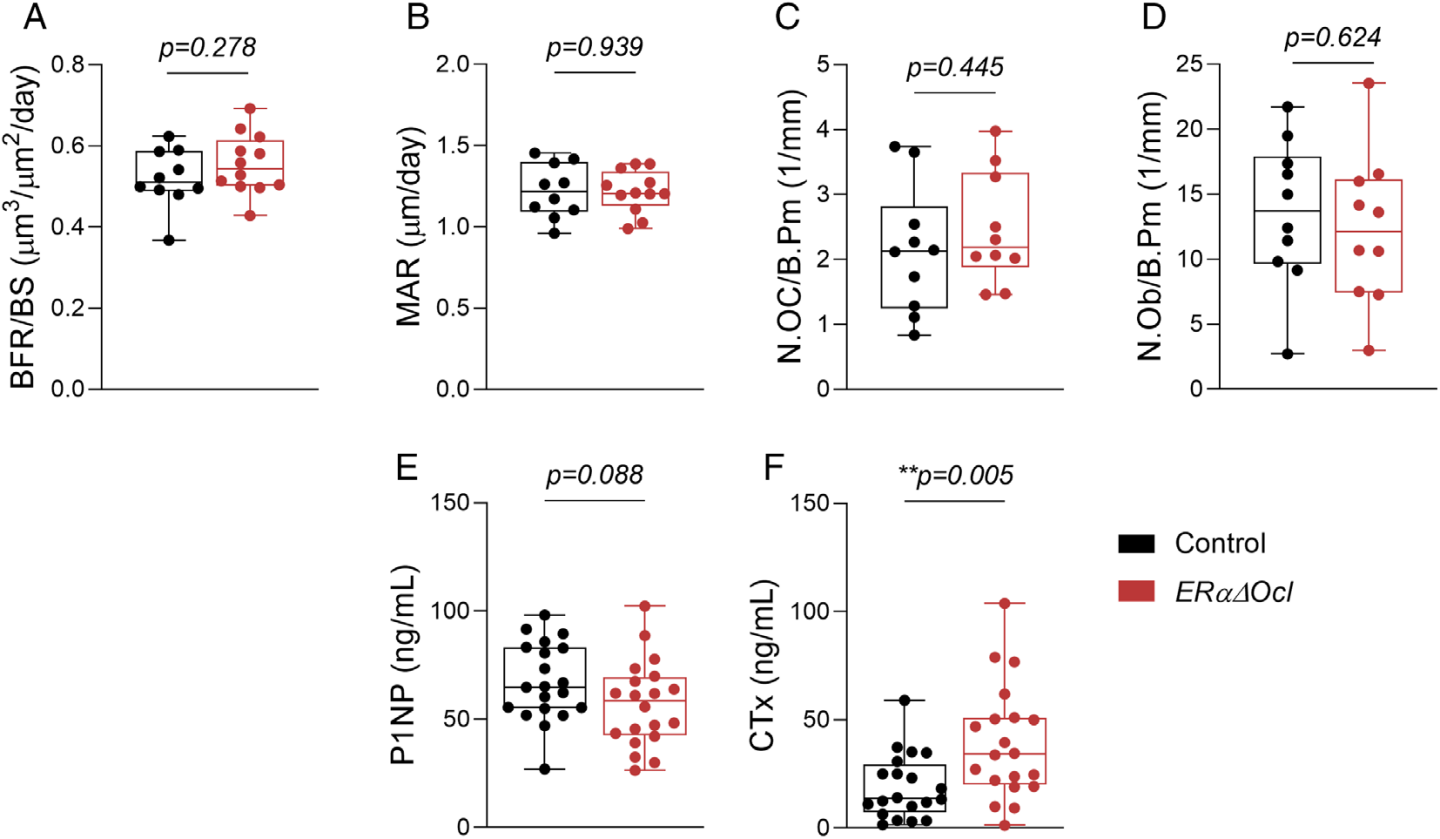
ERα deletion in osteoclasts does not affect bone formation or resorption but may increase osteoclast activity. (*A*) Measurement of bone formation rate per bone surface (BFR/BS) and (*B*) mineral apposition rates (MARs) in lumbar spines of female *ERαΔOcl* and control mice using double‐label dynamic histomorphometry. (*C*) Counted number of osteoclasts (N.OC/B.Pm) and (*D*) osteoblasts (N.OB/B.Pm) normalized to bone perimeter using static histomorphometry. (*E*, *F*) Dynamic histomorphometry measurements in female *ERαΔOcl* and control mice. (*F*–*H*) Osteoclast and osteoblast numbers in *ERαΔOcl* and control mice. Statistical significance determined by unpaired *t*‐test. (*A*–*D*) *n* = 10–12 per group, (E, F) *n* = 19–20 per group.

To investigate any alterations in bone formation or resorption that might explain the skeletal phenotype, we performed static and dynamic bone histomorphometry in *ERαΔOcl* mice. We focused on female mice where previous studies on noninducible osteoclast‐specific ERα deletion showed clear skeletal effects.^(^ ^8^, ^9^ ^)^ In the *ERαΔOcl* mice, there were no significant changes in bone formation (Fig. 4*A*) or mineral apposition (Fig. 4*B*) rates. Additionally, both osteoclast and osteoblast numbers were unchanged relative to control mice (Fig. 4*C*,*D*). Interestingly, although serum P1NP levels (bone formation) were unchanged (Fig. 4*F*), there was a significant increase in serum CTx levels (bone resorption) in the *ERαΔOcl* mice. This finding, in the context of a lack of changes in osteoclast numbers (Fig. 4*C*) and a trend toward decreased spine BV/TV (Fig. 3*A*), indicates that inducible *ERα* deletion results in an increase in osteoclast activity, but not in osteoclast numbers.

**Fig. 5:**
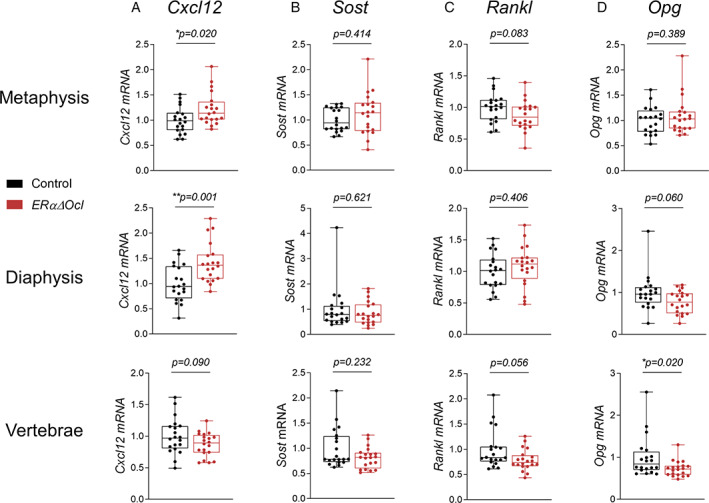
*ERαΔOcl* mice display increased *Cxcl12* expression yet exhibit no change in osteoblast‐derived bone turnover genes. mRNA expression of (*A*) *Cxcl12*, (*B*) *Sost*, (*C*) *Rankl*, and (*D*) *Opg* in *ERαΔOcl* mice (fold‐change relative to control group) at femur diaphysis, metaphysis, and thoracic spine. *n* = 20 mice per group. Statistical significance determined by unpaired *t*‐test.

Previous studies using constitutive *ERα* deletion in osteoblast lineage cells (using the Prx1‐Cre^(^ ^24^ ^)^) as well as inducible *ERα* deletion in osteocytes in our parallel model (*ERαΔOcy* ^(^ ^11^ ^)^) demonstrated significant increases in the pro‐osteoclastogenic cytokine, *Cxcl12* (*SDF‐1*),^(^ ^25^ ^)^ in the bones of *ERα* knockout mice. Of interest, we found increased *Cxcl12* mRNA levels in the femur metaphysis and diaphysis in *ERαΔOcl* mice (Fig. 5*A*). By contrast, *Sost*, which was markedly increased in the bones of the *ERαΔOcy* mice,^(^ ^11^ ^)^ was unchanged in *ERαΔOcl* mice (Fig. 5*B*). Notably, bone *Opg* mRNA levels were significantly reduced in the vertebrae of the *ERαΔOcl* mice, with a similar trend for *Rankl* (Fig. 5*C*,*D*).

## Discussion

In this study, we developed and validated a new tamoxifen‐inducible Cre model targeting osteoclasts, analogous to our previous inducible model targeting osteocytes.^(^ ^11^ ^)^ In adult mice, the *Ctsk‐CreERT2* appears to be highly specific for osteoclasts; importantly, Cre activation by tamoxifen at 4 months of age avoided issues related to *Ctsk* expression in mesenchymal cells in the groove of Ranvier^(^ ^26^ ^)^ or in periosteal cells^(^ ^27^ ^)^ that may occur during embryonic or early postnatal development.

As previously recognized by Kedlaya and colleagues for the 10‐kb *Dmp1‐CreERT2* model^(^ ^28^ ^)^ and by us for the 8‐kb *Dmp1‐CreERT2* mice,^(^ ^11^ ^)^ it is challenging to demonstrate deletion of target genes in inducible Cre models. Even though we used the Ai9 *TdTomato* reporter mice to demonstrate specificity for osteoclasts, reliance solely on reporter mice is problematic since gene deletions with a given Cre can vary substantially from one floxed gene to another, likely due to local chromatin structure around floxed alleles.^(^ ^29^ ^)^ In addition, given the relatively low abundance of osteoclasts within bone, assessing the extent of gene deletion using DNA rearrangement or mRNA levels in whole bone lacks sufficient sensitivity due to the overwhelming number of contaminating cells not expressing Cre. As such, similar to our previous study with the *ERαΔOcy* model,^(^ ^11^ ^)^ we used in situ hybridization (RNAScope) for the *ERα* transcript combined with an osteoclast‐specific transcript (*Oscar*)^(^ ^23^ ^)^ and demonstrated a modest (from 52.3% to 32.5%) but significant reduction in osteoclasts positive for *ERα*. Of note, this reduction in *ERα* + osteoclasts in the *ERαΔOcl* mice was remarkably similar to the reduction in *ERα* + osteocytes we previously demonstrated in our inducible osteocytic *ERα* deletion model (from 51.1% to 38.8%).^(^ ^11^ ^)^

Despite a very similar extent of *ERα* deletion, the skeletal phenotype of the *ERαΔOcl* mice differed substantially from that of the identically treated *ERαΔOcy* mice.^(^ ^11^ ^)^ We previously found that female *ERαΔOcy* mice had significant reductions in spine BV/TV (−20.1%) accompanied by decreased trabecular bone formation rates (−18.9%). The female *ERαΔOcy* mice also had periosteal and endocortical expansion, but preserved cortical thickness, consistent with the known effects of estrogen to inhibit periosteal apposition and promote endocortical formation.^(^ ^30^ ^)^ In addition, osteoclast numbers were increased in trabecular bone in the *ERαΔOcy* mice. By contrast, female *ERαΔOcl* mice had a minimal skeletal phenotype, with only a borderline reduction in spine BV/TV and no change in osteoclast numbers, although they did exhibit an increase in serum CTx levels, consistent with an increase in osteoclast activity. In males, both the *ERαΔOcy* and *ERαΔOcl* mice had fairly unremarkable skeletal phenotypes.

The phenotype of the inducible *ERαΔOcl* mice was also different from that of two previous constitutive osteoclast *ERα* deletion models using either *Ctsk‐Cre* ^(^ ^8^ ^)^ or *LysM‐Cre*.^(^ ^9^ ^)^ As summarized in Table 1, constitutive *Ctsk‐Cre‐*mediated *ERα* deletion in female, but not male, mice led to trabecular osteopenia and increased osteoclast numbers by 12 weeks of age, with no alterations in cortical bone.^(^ ^8^ ^)^ The phenotype of the mice with *LysM‐Cre‐*mediated *ERα* deletion was slightly different, in that neither trabecular osteopenia nor increases in osteoclast numbers were evident at 12 weeks, but they were present by 22 weeks of age.^(^ ^9^ ^)^

**Table 1:** Phenotypic comparison of inducible versus constitutive ERα deletion in osteocytes and osteoclasts

	Inducible ERα deletion (5 months)		Constitutive ERα deletion	
	ERαΔOcl	ERαΔOcy^(^ ^11^ ^)^	Ctsk‐Cre^(^ ^8^ ^)^	LysM‐Cre^(^ ^9^ ^)^
BV/TV (spine)	=	↓	↓ (12 weeks)	↓ (22 weeks)
N.Oc/B.Pm	=	↑	↑	↑
CTx (serum)	↑	=	ND	ND

*Note*: BV/TV = bone volume fraction; N.Oc/B.Pm = osteoclast number per bone perimeter; “=” = no change; ↑ = parameter increases; ↓ = parameter decreases; ND = parameter not determined.

Collectively, comparison of the skeletal phenotype of current inducible *ERαΔOcl* mice to that of the identically treated inducible *ErαΔOcy* mice, as well as the two constitutive osteoclast *ERα* models, is informative in several respects (Table 1). First, in adult mice with inducible *ERα* deletion, osteocytic *ERα* deletion clearly has more profound skeletal consequences than osteoclastic *ERα* deletion. Second, constitutive *ERα* deletion in osteoclasts from conception onward also has more profound skeletal consequences than inducible osteoclastic *ERα* deletion in adult mice studied over 1 month. There are several potential explanations for the discrepancies between the inducible and constitutive osteoclastic *ERα* deletion models. First, the effects of inducible osteoclastic *ERα* deletion may take longer to manifest than the 1‐month time frame of the current study. Consistent with this, at least in the *LysM‐Cre ERα* deletion model, there was no skeletal phenotype at 12 weeks of age, and the phenotype was only evident by 22 weeks of age.^(^ ^9^ ^)^ A second explanation may come from recent work showing that, in contrast to adult osteoclasts that are derived from hematopoietic stem cells, neonatal and early‐life osteoclasts appear to have a different origin and are derived from erythromyeloid progenitor (EMP) cells.^(^ ^31^ ^)^ Thus, noninducible osteoclastic ERα deletion may target an entirely different osteoclast population, possibly influencing skeletal development, as compared to inducible osteoclastic ERα deletion in adult mice, which simulates postmenopausal loss of estrogen signaling. Finally, it is also plausible and perhaps likely that the increased bone resorption in states of estrogen deficiency in adult mice is mainly caused by lack of ERα‐mediated suppression of pro‐resorptive factors (e.g., receptor activator of NF‐κB ligand) in mesenchymal skeletal cells rather than through direct actions on osteoclasts. Although we offer these explanations for the lack of a significant skeletal phenotype in the adult inducible *ERαΔOcl* mice, more studies are clearly needed to address these possibilities.

Our data also demonstrate that the inducible *Ctsk‐CreERT2* mouse model only targets osteoclastic cells on endosteal bone surfaces and not periosteal bone surfaces. This is in contrast to the constitutive *Ctsk‐Cre* mouse model, which clearly shows periosteal expression of Cre.^(^ ^8^ ^)^ These observations are consistent with the fact that the *Ctsk‐CreERT2* inducible model is not active during development, whereas the constitutive *Ctsk‐Cre* is active from conception onward, so this is a distinct advantage of the inducible model.

A compelling interpretation in examining the bone phenotypes of the two ERα inducible models can be drawn when considering the role of ERα in adult osteoclasts versus osteocytes, in terms of their role on osteoclasts themselves. As seen in the Results and briefly summarized in Table 1, in the inducible *ERαΔOcl* mice there is no effect on osteoclast number (N.Oc/B.Pm), but an increase in serum CTx is observed, whereas in the *ERαΔOcy* mice, the opposite is found. This suggests that the cellular pools of ERα in adult osteoclasts and osteocytes may function in different capacities, with the osteoclastic pool influencing osteoclast activity and the osteocytic pool influencing osteoclast number.

One of the limitations of this study is that the bone phenotype of the inducible *ERαΔOcl* mice was only measured at one time point (5 months of age), as it is possible that a more definitive phenotype would be observed in older mice. However, this time point was chosen to assess the potential phenotype in young adult mice and to mirror the experimental conditions of the *ERαΔOcy* mice,^(^ ^11^ ^)^ in an effort to directly compare the skeletal consequences of inducible ERα deletion in osteoclasts and osteocytes, respectively. Future experiments examining the effects of these deletions in older mice may uncover important data not observed in this study.

In summary, we describe the development and validation of a new, tamoxifen‐inducible *Ctsk‐CreERT2* model that may circumvent a number of potential confounders of constitutive osteoclast‐specific Cre models. When comparing these mice to analogous models with inducible osteocytic ERα deletion, as well as mice with constitutive osteoclastic ERα deletion, our data indicate that osteocytic ERα plays a more important role in regulating adult bone metabolism than osteoclastic ERα, at least in female mice. Our study also points to potential differences between deleting ERα (or potentially other genes) in osteoclasts from conception onward versus inducibly in adult mice.

## Author Contributions

**Madison L. Doolittle:** Conceptualization; data curation; formal analysis; investigation; methodology; writing – original draft; writing – review and editing. **Brittany Eckhardt:** Investigation; methodology; writing – review and editing. **Stephanie Vos:** Investigation; methodology; writing – review and editing. **Sarah Grain:** Investigation; methodology; writing – review and editing. **Jennifer L Rowsey:** Investigation; methodology; writing – review and editing. **Ming Ruan:** Investigation; methodology; writing – review and editing. **Dominik Saul:** Formal analysis; investigation; methodology; writing – review and editing. **Joshua N Farr:** Investigation; methodology; writing – review and editing. **Megan Weivoda:** Investigation; methodology; writing – review and editing. **Sundeep Khosla:** Conceptualization; funding acquisition; project administration; supervision; writing – original draft; writing – review and editing. **David G. Monroe:** Conceptualization; data curation; formal analysis; funding acquisition; investigation; methodology; project administration; supervision; writing – original draft; writing – review and editing.

## Disclosures

The authors have no relevant financial disclosures and no conflicts of interest.

### Peer Review

The peer review history for this article is available at https://www.webofscience.com/api/gateway/wos/peer-review/10.1002/jbm4.10797.

## Supporting information


**Data S1.** Supporting Information.

## Data Availability

The data that support the findings of this study are available from the corresponding author upon request.
