# Supplementary material for: Modest Effects of Osteoclast‐Specific ERα Deletion after Skeletal Maturity
Source: JBMR Plus. 2023 Jul 13;7(10):e10797. doi: 10.1002/jbm4.10797 (PMC10556268; doi:10.1002/jbm4.10797)

Supplementary Material

Modest Effects of Osteoclast-Specific ERα Deletion After Skeletal Maturity

Madison L. Doolittle, Ph.D.[1], Brittany A. Eckhardt, B.S.[1], Stephanie J. Vos, B.S.[1], Sarah Grain, B.S.[1], Jennifer L. Rowsey, B.S.[1], Ming Ruan, M.S.[1], Dominik Saul, M.D.[1, 3], Joshua N. Farr, Ph.D.[1], Megan M. Weivoda, Ph.D.[2], Sundeep Khosla, M.D.[1*], and David G. Monroe, Ph.D.[1*]

Supplementary Material Figure Legends

Supplementary Figure 1. The *Ctsk-CreERT2* mouse model targets endosteal osteoclasts. The *Ctsk-CreERT2* mice were crossed to Ai9 *TdTomato* mice and treated with tamoxifen, exactly as in Fig. 1. Sections from three independent mice were stained for TRAP5 activity via the fluorescence phosphatase substrate ELF97 (green) and counterstained with DAPI (blue). White signal indicates TdTomato expression on the endosteal bone surfaces. Magnification is at 20X and scale bars = 100 μm.

Supplementary Figure 2. Inducible ERα deletion in osteoclasts leads to mild changes in bone microarchitecture in adult male mice. (A-D) Micro-CT analyses of trabecular bone at the lumbar spine describing BV/TV, Tb.Th, Tb.N, and Tb.Sp. (E-I) Longitudinal Tib. Dia. micro-CT analyses of cortical bone showing % change between baseline and endpoint describing EC, Ct.Th, PC, Ct.vBMD, and Ct.Po. n=19-20 mice per group. Statistical significance determined by unpaired t-test.

Supplementary Table 1. Mouse QPCR primer sequences used in this study.

Gene Symbol	Forward Primer (5' to 3')	Reverse Primer (5' to 3')
Cxcl12 (SDF-1)	GCCAACGTCAAGCATCTGAAA	CAGCCGTGCAACAATCTGAA
Sost (Sclerostin)	ACTTGTGCACGCTGCCTTCT	TGACCTCTGTGGCATCATTCC
Tnfsf11 (Rankl)	GCTGGGACCTGCAAATAAGT	TTGCACAGAAAACATTACACCTG
Tnfrsf11b (Opg)	CCAAGAGCCCAGTGTTTCTT	CCAAGCCAGCCATTGTTAAT
Actb	AATCGTGCGTGACATCAAAGAG	GCCATCTCCTGCTCGAAGTC
Gapdh	GGGAAGCCCATCACCATCTT	GCCTCACCCCATTTGATGTT
Hprt	CGTGATTAGCGATGATGAACCA	TCCAAATCCTCGGCATAATGA
Tuba1a	GGTTCCCAAAGATGTCAATGCT	CAAACTGGATGGTACGCTTGGT
Tbp	CTTCACCAATGACTCCTATGACCC	CGCAGTTGTCCGTGGCTCTCTTA

Supplementary Fig 1

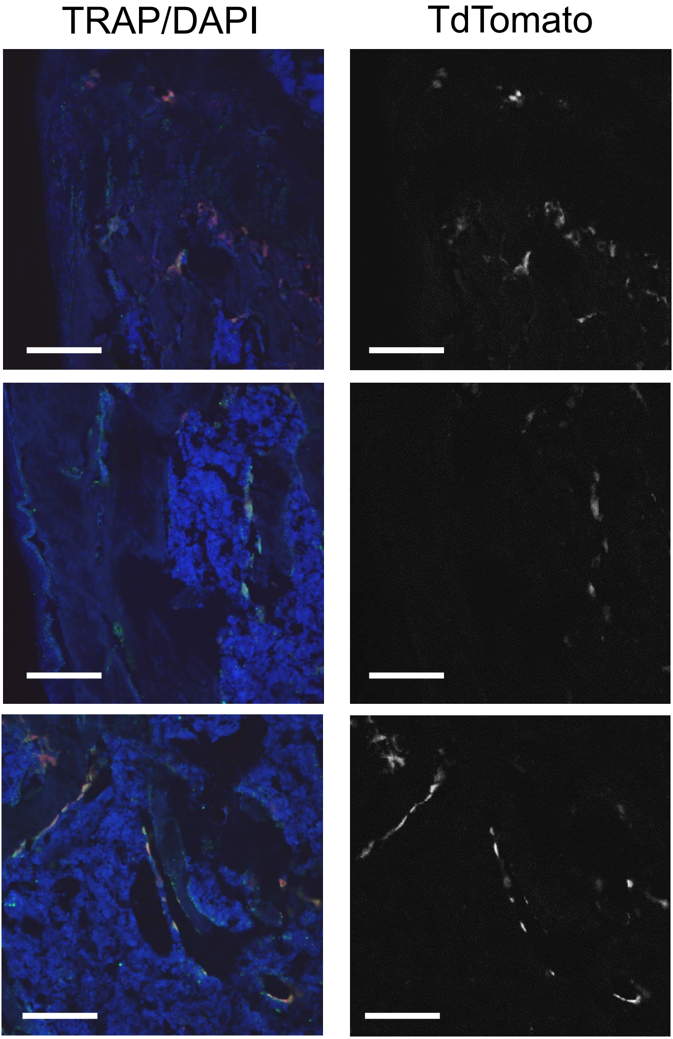

Supplementary Fig 2

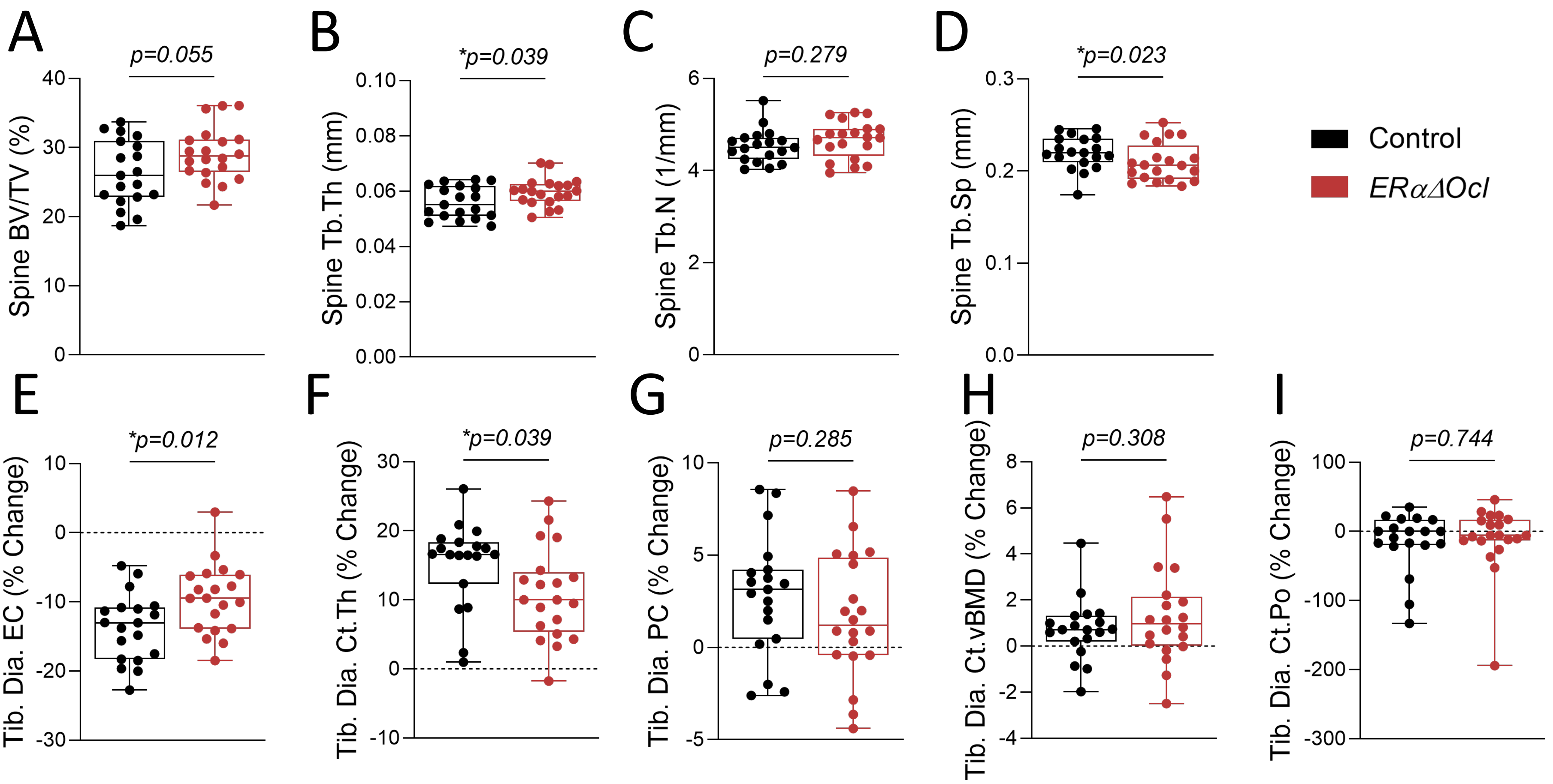

Supplement: Supplementary file 1 — Data S1. Supporting Information. [file JBM4-7-e10797-s001.pdf]
